# Reply to: “Does PCNA diffusion on DNA follow a rotation-coupled translation mechanism?”

**DOI:** 10.1038/s41467-020-18856-0

**Published:** 2020-10-05

**Authors:** Matteo De March, Silvia Onesti, Alfredo De Biasio

**Affiliations:** 1 Structural Biology Laboratory, Elettra-Sincrotrone Trieste S.C.p.A., Trieste, 34149 Italy; 2 Leicester Institute of Structural & Chemical Biology and Department of Molecular & Cell Biology, University of Leicester, Leicester, LE1 7HB UK

**Keywords:** Structural biology, DNA replication

**Replying to** Greenblatt et al. *Nature Communications* 10.1038/s41467-020-18855-1 (2020)

In their review, Greenblatt et al. criticise our model for the helical mechanism of PCNA sliding on dsDNA. Such helical sliding requires a certain pattern of interactions between the DNA phosphates and the positively charged residues on the PCNA inner rim, which we investigated by crystallography, NMR and atomistic MD simulations^1^. Greenblatt et al. agree that these PCNA–DNA interactions are (a) experimentally observed by NMR and (b) correctly predicted by MD simulations. However, the authors contest our crystallographic data and argue that our MD simulations are not conclusive.

Before arguing that our crystallographic data are correct, we would like to stress that helical sliding of PCNA is supported by previous single-molecule work by Van Oijen group^2^, suggesting that PCNA tracks the DNA helix 97–99% of the time while diffusing, consistent with a major rotation-coupled translation component in the sliding mechanism. Indeed, these data are also compatible with a minor translational component of PCNA sliding, uncoupled from the DNA helical pitch. In our paper^1^, we focus on the structural basis of the helical component, but we do not rule out the existence of alternative mechanisms of sliding.

Although macromolecular crystallography is becoming more and more of a black box, and a number of structures can be determined in a semi-automatic manner, biologically relevant information can often be obtained from medium to low-resolution data, where the degree of order and the intrinsic polymorphism of the structure require a manual, slow and careful approach, to ensure that the improvement of the model and the corresponding slow improvement of the phases provide the best chance for weak but significant features to emerge. Automated, quick refinement processes can fail to identify features that may have biological relevance. Partly disordered regions can be very sensitive to the exact choice of refinement programs, and refinement protocols. For example, we obtained the most informative electron density map after various attempts, by using REFMAC^3^, with TLS refinement and the use of an exponential (Babinet) bulk-solvent model instead of the mask-based flat bulk-solvent model. Moreover, a critical evaluation of crystallographic data cannot be done without an analysis of the biological context. The biological function of PCNA is to form transient and dynamic interactions with DNA. Not surprisingly, in the PCNA–DNA structure the DNA is not as well defined as the protein, and the corresponding electron density is weak and compatible with partial occupancy and/or the existence of a subpopulation of complexes with slightly different DNA orientations. We are aware of these limitations and we have discussed them in the paper^1^.

High DNA temperature factors are not unusual for proteins bound to DNA in a non-sequence-specific manner, thus establishing transient interactions that allow rapid diffusion of the protein along the nucleic acid. Among those are the structure of yeast PCNA bound to primed DNA (PDB-ID 3K4X^4^) where the DNA was also refined as a rigid body with TLS with high average DNA B-factors, especially when compared to the protein (285 vs 56 Å^2^). Numerous other examples include the histone-like protein HU (PDB-ID 4YEY, DNA B-factors 190 Å^2^)^5^; the UvrD helicase (PDB-ID 4C2T, DNA B-factors 250 Å^2^)^6^; the Ku heterodimer (PDB-ID 6ERF, DNA B-factors 250 Å^2^)^7^. From this far from an exhaustive list, it is clear that although the B-factors of the dsDNA in our structure are high, they are not unusually high. In the context of the PCNA functional role (sliding along the DNA and therefore establishing transient and highly dynamic contacts), it is not very surprising that the B-factors of both the DNA and residues involved in the interaction are not lower.

Greenblatt et al. claim to calculate an OMIT map using our deposited data (PDB-ID 6GIS), but this is not what they actually do. Instead, they remove a large and partly disordered chunk of the model (the DNA) and carry out simulated annealing refinement. The correct procedure to calculate an OMIT map is described by Terwilliger et al.^8^, in which density from a set of overlapping OMIT regions can be combined to create a composite iterative-built OMIT map that is ‘essentially free from the model bias yet that benefits from the power of iterative model building and refinement’. When we use this procedure, as implemented in Phenix^9^ as *composite OMIT map*, we obtain a clear indication that the DNA is indeed bound to the ring in a position that is consistent with our model (Fig. 1a). Phenix also offers the option of carrying out additional steps such as minimisation and simulated annealing, but the authors warn that these procedures are more ‘aggressive’ and may be problematic for difficult or low-resolution structures.

**Fig. 1:**
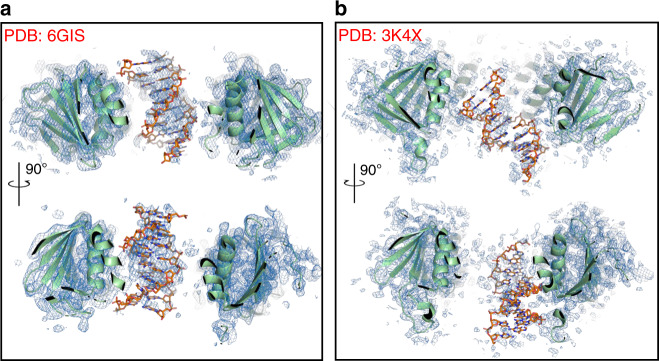
PCNA–DNA complexes OMIT maps. **a** Two views of the composite OMIT map of the human PCNA–DNA complex (PDB-ID 6GIS)^1^ contoured at 0.9 σ showing the PCNA central channel. PCNA is shown as a ribbon, DNA as sticks. **b** Two views of the composite OMIT map of the yeast PCNA–DNA complex (PDB-ID 3K4X)^4^ contoured at 0.9 σ.

As for the use of a Polder omit map^10^, again the Phenix documentation suggests as typical omitted regions ligands, surface residues, C- or N-terminal residues, and explicitly warns against omitting a large portion of the model at once, as the excluded bulk-solvent volume might become too large. All the examples described in the manuscript^10^ are indeed related to small-molecule ligands. We, therefore, feel that this procedure cannot be legitimately applied to our case.

Greenblatt et al. show the crystallographic map of the bacterial beta clamp bound to primed DNA (PDB-ID 3BEP), solved by O’Donnell group^11^, pointing at the stronger DNA density in the clamp channel. We believe this is not the correct example to report in this case, as in this structure the single-stranded portion of the DNA is bound to the clamp in a mode incompatible with sliding. A more suitable example is the crystal structure of yeast PCNA bound to primed DNA, solved by O’Donnell and Kuriyan groups^4^. We calculated a composite OMIT map using the deposited structure factors (PDB-ID 3K4X) (Fig. 1b), showing that the resulting density ascribed to DNA is weaker and noisier than the one in our structure (Fig. 1a). Nonetheless, the yeast PCNA–DNA structure has never been questioned and has provided useful biological insights.

To summarise, our X-ray derived model of the human PCNA–DNA complex, despite some limitations inherent to the system, is plausible, in agreement with our NMR data regarding the site of binding of the DNA, and correctly recapitulated by our atomistic MD simulations^1^. Together, our data attempt to explain the helical component of PCNA sliding. A full molecular description of the complex behaviour of stand-alone PCNA sliding on dsDNA^2^ requires further investigation. While we deem such investigation interesting, we want to stress that the real challenge in understanding the biology of PCNA is the description of the functional interaction of PCNA and DNA in the presence of physiological PCNA binding partners, such as DNA polymerases.

## Data Availability

Data supporting the findings of this study are available from the corresponding author upon reasonable request.
